# Making Home Care Work for All: Real-World Considerations for Effective, Affordable Care Policies

**DOI:** 10.1093/geroni/igaf122.1440

**Published:** 2025-12-31

**Authors:** Jennifer Reckrey, Emily Franzosa, Joanne Spetz

## Abstract

Older adults increasingly rely on paid care from home health aides and other home care workers to age in place. There is broad agreement that home care is too costly for most families and pay is too low for home care workers, jeopardizing access to these vital services. However, there is less consensus on the most impactful policy solutions to make home care more effective and affordable. This symposium explores how cost and finances shape paid care delivery and highlights successes and challenges of models that aim to address financial barriers to care. First, Miller et al share a study examining support for paid caregiving policy across political affiliations, finding bipartisan support for expanding financial access to affordable home care and increasing the capacity of the paid caregiving workforce. Reckrey and Shen then use data from the Health and Retirement Study to illustrate the limited ability of most people living with dementia to pay for needed home care after diagnosis. Franzosa and colleagues examine home care within the integrated Veterans Health Administration, finding that even with minimal or no co-payments, means testing and administrative burden can create access barriers. Finally, Angell et al discuss how well-intentioned wage increases for direct care workers may reduce their eligibility for public benefits, erasing financial gains. Taken together, these presentations highlight the complexity of policies seeking to improve access to paid care and highlight the need for nuanced approaches to ensure affordable access to these critical services for those who need them most. Paid Caregiving Interest Group Sponsored Symposium

